# Fasting in diabetes treatment (FIT) trial: study protocol for a randomised, controlled, assessor-blinded intervention trial on the effects of intermittent use of a fasting-mimicking diet in patients with type 2 diabetes

**DOI:** 10.1186/s12902-020-00576-7

**Published:** 2020-06-24

**Authors:** Elske L. van den Burg, Marjolein P. Schoonakker, Petra G. van Peet, M. Elske. van den Akker-van Marle, Ko Willems van Dijk, Valter D. Longo, Hildo J. Lamb, Mattijs E. Numans, Hanno Pijl

**Affiliations:** 1 Department of Public Health and Primary Care, Leiden University Medical Center (LUMC), Postzone V0-P, Postbus 9600, 2300 RC, Leiden, The Netherlands; 2 Department of Biomedical Data Sciences, Medical Decision Making, Leiden University Medical Center, Leiden, the Netherlands; 3 Internal Medicine, Leiden University Medical Center, Leiden, the Netherlands; 4 Human Genetics, Leiden University Medical Center, Leiden, the Netherlands; 5 FIRC Institute of Molecular Oncology, Milan, Italy; 6 Longevity Institute, Davis School of Gerontology, University of Southern California, Los Angeles, USA; 7 Radiology, Leiden University Medical Center, Leiden, the Netherlands

**Keywords:** Diabetes mellitus, type 2, Glycated hemoglobin a, Diet therapy, Fasting, Fasting-mimicking diet, Cost-effectiveness

## Abstract

**Background:**

Caloric restriction is an effective way to treat Type 2 diabetes (T2D). However, chronic and severe restriction of food intake is difficult to sustain and is known to promote slower metabolism. Intermittent and frequent fasting can exert similar metabolic effects, but may be even more challenging for most patients. A fasting-mimicking diet (FMD) is low in calories, sugars and proteins, but includes relatively high levels of plant based complex carbohydrates and healthy fats. The metabolic effects of such a diet mimic the benefits of water-only fasting. The effects of a FMD applied periodically in T2D patients are still unknown. The Fasting In diabetes Treatment (FIT) trial was designed to determine the effect of intermittent use (5 consecutive days a month during a year) of a FMD in T2D patients on metabolic parameters and T2D medication use compared to usual care.

**Methods:**

One hundred T2D patients from general practices in the Netherlands with a BMI ≥ 27 kg/m^2^, treated with lifestyle advice only or lifestyle advice plus metformin, will be randomised to receive the FMD plus usual care or usual care only. Primary outcomes are HbA1c and T2D medication dosage. Secondary outcomes are anthropometrics, blood pressure, plasma lipid profiles, quality of life, treatment satisfaction, metabolomics, microbiome composition, MRI data including cardiac function, fat distribution and ectopic fat storage, cost-effectiveness, and feasibility in clinical practice.

**Discussion:**

This study will establish whether monthly 5-day cycles of a FMD during a year improve metabolic parameters and/or reduce the need for medication in T2D. Furthermore, additional health benefits and the feasibility in clinical practice will be measured and a cost-effectiveness evaluation will be performed.

**Trial registration:**

The trial was registered on ClinicalTrials.gov. Identifier: NCT03811587. Registered 21th of January, 2019; retrospectively registered.

## Background

Type 2 diabetes (T2D) is a multifactorial disease, emanating from gene-environment interactions [1]. Diet quality and quantity are at the heart of its pathogenesis [2]. A positive energy balance, often caused by an excess of carbohydrates, leads to insulin resistance, impaired insulin secretion and hyperglycaemia [3–5]. Currently available medication for T2D influences glucose metabolism, but does not address the cause of T2D. In contrast, lifestyle interventions including dietary strategies can redress the underlying pathogenic mechanisms.

Caloric restriction and weight loss influence the course of T2D in a positive way. A loss of 5% of bodyweight or more reduces glycated haemoglobin (HbA1c), lipoprotein levels, and blood pressure [6]. Moreover, several studies have shown that T2D is not necessarily a chronic progressive disease and that remission can be achieved by continuous caloric restriction [7–9]. However, restriction of calories for a longer period of time can be difficult to sustain [10]. Prolonged caloric restriction has also been shown to reduce basal metabolic rate, even when adjusted for body weight reduction, indicating that continuous caloric restriction not only causes weight and lean body mass loss, but can also cause metabolic changes that may make it very difficult to reach and maintain a healthy weight and fat mass [11].

Preclinical experiments have shown various health benefits of intermittent and periodic fasting. For example, alternate day fasting (ADF) in mice reduces serum glucose and insulin levels and improves glucose tolerance [12, 13]. Human studies reveal similar effects, indicating that intermittent and frequent fasting can reduce calorie intake and fat mass [14], and decrease fasting glucose in patients with T2D [15, 16]. The health benefits of fasting are caused by several molecular mechanisms, including the reduction of ectopic fat storage, insulin levels, endogenous glucose production and IGF-1 [17]. Furthermore, it increases adipose lipolysis and fat oxidation, and glycerol and ketone bodies instead of glucose are used as preferred carbon sources. However, intermittent water-only fasting may be very difficult for patients to adhere to for long periods of time. Moreover, prolonged and frequent fasting may exacerbate pre-existing nutritional deficiencies, which may be unsafe for frail individuals [17], in addition to being associated with other potential risks in those extending the fasting period by skipping breakfast daily [18, 19].

Fasting-mimicking diets (FMDs) have been developed to mimic the endocrine and metabolic effects of complete fasting, while providing a modest number of calories [17]. To this end, FMDs comprise virtually no refined sugar or starch, low protein levels, complex carbohydrates and healthy fats, all from plant based sources [20–22]. In a pilot study, 38 healthy adults were randomised between three monthly, five-day cycles of FMD, or continuation of their normal diet; the FMD caused weight loss and reduced visceral fat [20]. In a larger study of 1 hundred healthy participants, three monthly, five-day FMD cycles reduced body weight, trunk and total body fat, lowered blood pressure and decreased IGF-1 [21]. A post hoc analysis showed a significant reduction in body mass index (BMI), blood pressure, triglycerides, and total and low-density lipoprotein cholesterol. In addition, in participants with an increased fasting glucose level (defined as 5.5 mmol/L or higher), the three FMD cycles brought glucose within the healthy range. The FMD was well tolerated without significant side effects [20, 21].

The above mentioned results suggest that a FMD could potentially be beneficial for patients with T2D, while minimizing the burden of frequent water-only fasting as well as side effects, such as slowing metabolism and loss of lean body mass. The FMD used in the Fasting In diabetes Treatment (FIT) trial is a five-day program providing patients with all the meals and containing ~ 1100 kcal on the first day and ~ 750 kcal on the following days [20, 21]. We will examine whether monthly five-day cycles of a FMD during a year improve metabolic parameters and/or reduce the need for medication in people with T2D treated by their General Practitioner (GP). We will also evaluate anthropometrics, blood pressure, plasma lipid profiles, quality of life, treatment satisfaction, metabolomics, microbiome composition and MRI data including cardiac function, fat distribution and ectopic fat storage. Furthermore, a cost-effectiveness evaluation will be performed and the feasibility in clinical practice will be measured. In the FIT trial, 1 hundred T2D patients from general practices in the Netherlands with a BMI ≥ 27 kg/m^2^, treated with lifestyle advice only or lifestyle advice plus metformin, will be randomised to receive the FMD plus usual care or usual care only. To our knowledge, we are the first to examine the impact of a FMD on a variety of health parameters in people with type 2 diabetes mellitus, who are treated by their GP.

### Primary study objective

- To evaluate the effect of the intermittent use (5 consecutive days a month during a year) of a FMD on the combined primary outcomes HbA1c and antidiabetic medication dosage in T2D patients compared to usual care.

### Secondary study objectives

- To evaluate the effect of the intermittent use (5 consecutive days a month during a year) of a FMD on anthropometrics, blood pressure, plasma lipid profiles, metabolomics, microbiome, MRI data (e.g. cardiac function and fat distribution), change in dietary pattern and physical activity in T2D patients compared to usual care.
- To perform a cost-effectiveness evaluation comparing the FMD with usual care.
- To evaluate the effects on quality of life and treatment satisfaction between the FMD group and the usual care group.
- To evaluate the feasibility in practice, measured as the percentage of compliant patients in the FMD group.

## Methods and design

### Study design

The study is designed as a randomised, controlled, assessor-blinded intervention trial on the effects of the intermittent use of a fasting-mimicking diet in T2D patients regularly treated in primary care. The trial is conducted in the Leiden University Medical Center (LUMC) in the Netherlands, according to the principles of the Declaration of Helsinki in accordance with the Medical Research Involving Human Subjects Act (WMO) and to the standards of Good Clinical Practice (GCP). The study was approved by the Medical Research Ethics Committee of the LUMC.

### Recruitment

Participants will be recruited from general practices in the area around Leiden and The Hague. General practice centres will be invited to collaborate in the study by contacting them through several care groups in the region of the province of South Holland. Potential participants will be identified by a computerised search of the general practices’ records. Lists of possibly eligible patients generated by the search will be reviewed by the GP and/or a physician assistant. Individuals likely to be ineligible, or unsuitable to approach because of co-morbidity or other practical or medical obstacles to participation, will be removed from this list. Potentially eligible participants will receive a patient information letter from their GP sent by regular mail with an invitation to participate in the study. Permission to disclose identifying data is given by sending back a response-leaflet to the investigator and/or the GP, using reply-paid envelopes. The GP and/or the physician assistant have the liberty to remind non-responders once.

Following agreement and disclosure of identifying data, potential participants will receive additional information from the investigators. When still interested in participation, an appointment for the first visit will be made, where the study will be fully explained and discussed. After written informed consent has been obtained, the participant will be screened further for eligibility.

Patients with T2D who hear from the study through other routes than the ones stated above and wish to participate, can participate if they fulfil the inclusion criteria and their GP does not have any objections. The inclusion period will last approximately 1,5–2 years, counting from the first inclusion.

If it is not possible to recruit enough participants through the various care groups, we can pre-select potential candidates in the anonymous routine healthcare database “ELAN data warehouse”. GPs in the Leiden/The Hague region share their anonymized patient data in this database. The ELAN data warehouse will then be used to preselect potential study participants from GPs who collaborate with us in this study. These GPs will identify and invite potential participants by matching electronic medical record code numbers.

### Study population

One hundred individuals (men or women) with T2D and a BMI ≥ 27 kg/m^2^, who are treated with lifestyle advice only and have a HbA1c > 48 mmol/mol, or who are treated with the combination of lifestyle advice and metformin, will be recruited for the study.

### Inclusion criteria

In order to be eligible to participate in the study, a participant must meet the following criteria:

- Diagnosis of type 2 diabetes
- Age > 18 years and < 75 years
- BMI ≥ 27 kg/m^2^
- Treatment with lifestyle advice and meeting at least one of the following two criteria:
  - HbA1c > 48 mmol/mol
  - Treatment with metformin

### Exclusion criteria

A potential participant who meets any of the following criteria will be excluded from participation in this study:

- Recent myocardial infarction (< 6 months)
- Creatinine clearance < 30 ml/min/1,73m^2^
- Pregnancy
- Contraindications for Magnetic Resonance Imaging (MRI)
- Allergy for nuts, sesame, soy or another ingredient of the diet
- History of syncope with calorie restriction in the past
- Any significant other disease (at the discretion of the investigator)

### Screening

At screening for eligibility, medical history will be taken and a physical exam will be performed to collect information on in- and exclusion criteria. Fasting blood levels will be recorded for HbA1c, glucose, creatinine, aspartate aminotransferase (ASAT), alanine aminotransferase (ALAT), haemoglobin, red cell indices, leucocytes, thrombocytes. If it is unclear whether someone is eligible for inclusion, patient information can be requested from the GP, other specialists or the pharmacy.

### Intervention

Participants will be randomised to receive the FMD for 5 consecutive days a month during a year plus usual care or usual care only. The use of metformin will be continued during the study, including the FMD periods.

### The fasting-mimicking diet

The FMD is a meal replacement plan that mimics water-only fasting. It consists of ingredients that are generally regarded safe and is completely plant based. It consists of bars, soups and beverages aiming to achieve a consistent and effective short-term calorie restriction, while providing adequate micronutrients. An example of the meal plan can be found in Table 1. Very low protein and sugar content is essential for this diet. Complex carbohydrates and polyunsaturated fatty acids are its main components. It entails a 5 day regimen. For patients who weigh less than 100 kg, day 1 has ~ 1100 kcal (10% protein, 56% fat and 34% complex carbohydrate); days 2–5 are identical and provide ~ 750 kcal (9% protein, 44% fat, 47% complex carbohydrate). For patients who weigh more than 100 kg, the diet will be amplified with one bar a day (90 kcal) with similar characteristics as the rest of the diet. The diet is lactose and gluten free, but contains nuts, soy and tomato as ingredients. Water and herbal tea can be consumed without a limit during the 5 days of the diet. Coffee or tea containing caffeine are limited to one cup a day. All items to be consumed per day are placed in an individual package, which will prevent the participant from consuming components of one of the following days by accident.

**Table 1:** Example meal plan of the fasting-mimicking diet for study participants

	Day 1	Day 2	Day 3	Day 4	Day 5
**Breakfast**	Tea	Tea	Tea	Tea	Tea
	Nut bar	Nut bar	Nut bar	Nut bar	Nut bar
	Algal Oil capsule				Algal Oil capsule
**Lunch**		Tea	Tea	Tea	Tea
	Tomato Soup	Mushroom Soup	Tomato Soup	Vegetable Soup	Tomato Soup
	Olives	Olives	Kale Crackers	Olives	Kale Crackers
	Kale crackers				
	Vitamin capsule	Vitamin capsule	Vitamin capsule	Vitamin capsule	Vitamin capsule
**Afternoon**	Tea	Tea	Tea	Tea	Tea
	Nut bar	Olives		Olives	
**Dinner**		Tea	Tea	Tea	Tea
	Minestrone Soup	Quinoa Mix Soup	Minestrone Soup	Quinoa Mix Soup	Minestrone Soup
	Choco crisp bar	Choco crisp bar		Choco crisp bar	
	Vitamin capsule	Vitamin capsule	Vitamin capsule	Vitamin capsule	Vitamin capsule
		Syrup for water flavouring	Syrup for water flavouring	Syrup for water flavouring	Syrup for water flavouring

### Usual care

To evaluate the feasibility of the use of the FMD in clinical practice, patients will be controlled by their general practitioner as usual and treated according to the Dutch treatment guideline for T2D for GP’s [23]. Medication dosage can be adapted by the GP as required to attain an HbA1c of 53 mmol/mol. If at any moment during the trial HbA1c is lower or higher than this target value, metformin (or any other prescription drug to reduce plasma glucose concentration) will be stopped, continued or increased at the discretion of the GP.

### Randomisation and treatment allocation

Randomisation will be performed after written informed consent and screening, when a participant is eligible based on the eligibility criteria. Participants will be randomised to receive the FMD in addition to usual care or usual care only. To ensure equal allocation of subgroups, stratification will be performed for gender and for weight < 100 kg and > 100 kg at baseline. Randomisation will be performed using permuted block randomization with blocks sizes 2 and 4.

Treatment allocation is computer generated via the electronic trial database in Castor EDC [24]. Allocation sequence is concealed by Castor EDC until randomisation of a participant. Castor EDC is used to assign participants to the different groups.

### Blinding

Because this is a dietary intervention study, it is not possible for participants or all study personnel to be blinded to group assignment. The study will be assessor-blinded, as the study research nurses who will perform the data collection (e.g. anthropometrics and blood pressure), and the technicians measuring gut microbiota and laboratory works will be blinded to intervention assignment. Moreover, the MRI images will be presented to the investigator anonymously (study identification number only) for analysis.

### Data collection

**Fig. 1:**
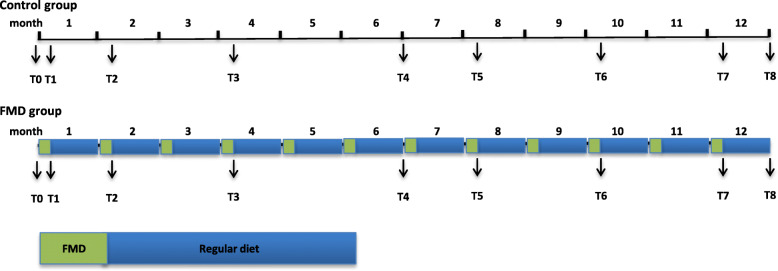
Timeline of assessments from T0 (baseline) to T8, also in relation to the timing of the FMD

Data collection will be performed by study research nurses at baseline and follow-up visits at the LUMC. All study nurses have received training on how to perform the data collection during the study visits. Follow-up time is 1 year. Participants come in for more extensive visits at study baseline, after 6 months and after 12 months. Furthermore, participants are seen several times in between the extensive study visits. For a detailed timeline of assessments, see Fig. 1. For details on the assessments during the study visits, see Table 2. Participants in the FMD group will receive a telephone call from the researchers during the FMD period, to promote participant compliance to the diet. If applicable, adverse events are recorded.

**Table 2:** Detailed schedule of assessments during follow up

Follow up	Screening	Baseline	In the first month	In the second month	In the fourth month	After six months	In the eighth month	In the tenth month	In the twelfth month	After twelve months
		T0	T1	T2	T3	T4	T5	T6	T7	T8
**Participant characteristics**	X									
**Medical history**	X									
**Anthropometrics**	X	X	X	X	X	X	X	X	X	X
**Blood pressure**	X	X	X	X	X	X	X	X	X	X
**Medication dosage**	X	X				X				X
**Adverse Events**		X				X				X
**Serious Adverse Events**		X	X	X	X	X	X	X	X	X
**MRI**		X				X				X
**Stool sample (including registration form)**		X	X			X				X
**Finger-stick ketone bodies test**		X	X		X		X		X	
**Questionnaires**										
Cost questionnaire					X	X		X		X
Quality of Life (EQ-5D-5L)		X			X	X		X		X
Treatment satisfaction (DTSQs)		X			X	X		X		X
Treatment satisfaction (DTSQc)										X
Eating habits (DHD-FFQ)		X				X				X
Physical activity (SQUASH)		X				X				X
MRI safety questionnaire	X	X				X				X
**Fasting blood sample**										
HbA1c	X	X				X				X
Glucose	X	X	X		X	X	X		X	X
Creatinin	X									
ASAT	X									
ALAT	X									
Haemoglobin	X									
Red cell indices	X									
Leucocytes	X									
Thrombocytes	X									
OGTT (5x glucose and 5x insulin)		X				X				X
Total cholesterol		X				X				X
Low-density lipoprotein (LDL)		X				X				X
High-density lipoprotein (HDL)		X				X				X
Triglycerides		X				X				X
High sensitive CRP		X				X				X
Metabolomics		X								X
IGF-1^a^		X	X		X		X		X	
Insulin-like growth factor-binding protein (IGF-BP-1)^a^		X	X		X		X		X	

Laboratory tests with a ^a^ will only be performed in the FMD group

In compliance with the GCP guidelines, the investigator will store all source documents that support the data collected from each subject, as well as all essential study documents. Source documents include paper case report forms (CRFs), Castor EDC (eCRFs) and Hix ChipSoft (Electronic Health Records Software used in the LUMC). Participant specific data will be labelled with a study identification number and entered in the electronic trial database in Castor EDC. The study identification number will be linked to personal information of the participant used for trial logistics in a separate electronic study database, which will be safeguarded by the investigator in accordance with GCP rules.

### Outcomes

An overview of the outcomes measured at each stage of the FIT study can be found in Table 2. Height will be measured in cm. Body weight will be measured to the nearest 100 g in light clothing without shoes. Body fat percentage by Bioelectrical Impedance Analysis will be performed in the same conditions. Waist circumference will be measured in cm halfway between the point of the lowest rib and the iliac crest. Blood pressure will be measured with participants seated, an average of two measurements will be taken. For an overview of the different questionnaires used in the FIT study, see Table 2. Participants will be requested to bring a recent overview of their medication use from their GP or their pharmacy.

Finger-stick test for measurement of ketone bodies will be performed in fasting conditions. Blood will be collected in fasting conditions, for details of laboratory tests, see Table 2.

The oral glucose tolerance test (OGTT) will be performed at baseline, after 6 months and after 12 months. Participants will be asked to stop using all glucose lowering medication for 1 day prior to any OGTT. A Venflon catheter will be inserted into an antecubital vein. Participants will be asked to drink a standard solution of 75 g of glucose dissolved in 300 ml of water. Blood will be collected at time 0, 30, 60, 90 and 120 min for measurement of glucose and insulin (at all timepoints).

MRI will be performed after at least 6 hrs of water-only fasting, after verifying that there are no contraindications to perform an MRI. The MRI will take place at baseline, after 6 months and after 12 months. All imaging will be performed on an MRI system operating at a field strength of 3 Tesla (Philips Medical Systems, Eindhoven, The Netherlands). Total scan time will be around 60 min. No additional contrast or medication will be administered during the examination. Cardiovascular MRI will be performed, for which a vector electrocardiogram and a breathing registration sensor is positioned on the abdomen, and a cardiac coil is placed over the chest. Breathing instructions are given through a headset. Multiple scans will be performed to determine heart dimensions and systolic and diastolic function of the heart. Flow through the mitral, tricuspid, aortic, and pulmonary valves will be assessed. For measuring body fat distribution, the volumes of the visceral and subcutaneous fat depositions of the body trunk will be visualized and quantified. Fat distribution in liver (LiverMultiScan, Perspectum Diagnostics, Oxford, UK) and heart tissue (Custom made software patch for respiratory navigator gated 1H-MR Spectroscopy, Leiden, The Netherlands) will be determined.

Stool samples will be collected at home using provided equipment and instructions. Samples will be stored at home in their refrigerator and brought to clinic within max 2 days. After receiving the stool samples, the samples will be processed and stored at − 80 °C until further analysis.

### Primary outcome

The primary endpoints of the study are HbA1c and antidiabetic medication dosage. These primary endpoints will be combined to compare the total successful outcomes between the FMD group and the control group after 12 months of follow up. A successful outcome will be defined as any reduction in the antidiabetic medication dosage and/or a decrease of HbA1c of 5 mmol/mol or more. HbA1c and antidiabetic medication dosage will also be recorded after 6 months.

### Secondary outcomes

- Bodyweight, waist circumference, body mass index and body fat percentage: recorded at baseline and every following study visit.
- Blood pressure: systolic and diastolic values measured at baseline and every following study visit.
- Fasting glucose: measured at baseline, after 6 months and after 12 months.
- Glucose and insulin response to an oral glucose load: OGTT measured at baseline, after 6 months and after 12 months.
- Plasma lipid profiles: measured at baseline, after 6 months and after 12 months.
- Metabolomics: measured at baseline and after 12 months.
- High sensitive CRP: measured at baseline, after 6 months and after 12 months.
- Dosage of statins and antihypertensive medication: recorded at baseline, after 6 months and after 12 months.
- Visceral fat, liver/heart ectopic fat, total body fat: from magnetic resonance imaging, measured at baseline, after 6 months and after 12 months.
- Cardiovascular function: from magnetic resonance imaging, measured at baseline, after 6 months and after 12 months.
- Gut microbiota architecture: measured from stool samples stored at baseline, after 1 week, after 6 months and after 12 months.
- Change in dietary pattern: DHD-FFQ measured at baseline, after 6 months and after 12 months [25].
- Cost-effectiveness analysis: The economic evaluation will consist of both a trial-based cost-effectiveness analysis with a time horizon of 12 months and a model-based cost-utility analysis with a lifetime horizon.
- Change in physical activity: SQUASH measured at baseline, after 6 months and after 12 months [26].
- Quality of life: EQ-5D-5L measured at baseline, after 3 months, after 6 months, after 9 months and after 12 months [27, 28].
- Treatment satisfaction: DTSQs at baseline, after 3 months, after 6 months, after 9 months and after 12 months [29]. DTSQc after 12 months [30].
- Feasibility in clinical practice: Percentage of fully compliant participants after 12 months, where compliance is based on self-report and laboratory measures. Glucose levels and finger-stick ketone bodies tests are performed after several FMD periods.

### Power calculation

The number of participants that will be included in the study is limited to 100 (50 in each group). We computed expected effect sizes with a sample size of 100. As all participants will be asked to come in for T8, we work with a limited loss to follow-up of 10% [8], thus basing the calculations on 90 participants (45 in each group).

We computed the effect size that can be detected with 80% power for the binary endpoint, which is equal to one if either a lower dose of antidiabetic medication is used at the end of the study compared to the start, if HbA1c is at least 5 mmol/mol lower at the end compared to study start, or both.

The expected percentage of participants in the control group who will reach this endpoint is between 4 and 6% [8, 31]. For this power calculation, we assumed a percentage of 5% of participants in the control group who will reach the combined endpoint. With a significance level of 5 and 80% power, using a two-sided binomial test with 45 participants in each group, an absolute difference in percentages of 21% (i.e. 5% in the control group and 26% in the treatment group) can be detected.

### Statistical analysis

Data will be analysed on an intention-to-treat basis at the 12-month point. In addition, a per protocol analysis well be carried out. A significance level of 5% will be used.

### Baseline data

Baseline data from the two groups will be compared on the following baseline values: age, gender, BMI and metabolic parameters. The values will be summarized using mean and standard deviation, or in case of an asymmetric distribution, by using median and interquartile range.

Hypothesis tests will be carried out using t-tests for independent samples, or, if the assumption of normality is violated, with a Mann-Whitney test. Multiple testing will be corrected for by using the Benjamini-Hochberg procedure. If the groups are found to be imbalanced on one or more covariates, then the following analyses will be adjusted for those covariates. Furthermore, the following analyses will be adjusted for gender and for weight < 100 kg or > 100 kg, since randomization is stratified for these values.

### Primary and secondary outcomes

Primary and secondary outcomes will be summarized using mean and standard deviation, or in case of an asymmetric distribution, by using median and interquartile range. HbA1c and antidiabetic medication dosage will be compared between the two groups, using t-tests for independent samples, or, if the assumption of normality is violated, with a Mann-Whitney U test. The values at 12 months will be used for these tests.

To compare the total successful outcomes, the primary endpoints HbA1c and antidiabetic medication dosage will be combined. A successful outcome will be defined as any reduction in the antidiabetic medication dosage and/or a decrease of HbA1c of 5 mmol/mol or more. This will be compared between the two groups as described above. To correct for multiple testing, the procedure of Benjamini-Hochberg will be used.

To detect a difference in the profiles of the outcomes that are measured repeatedly during the course of the study, a mixed model will be used. This model can be used if there is missing data, and if participants have their visits at different time points. The interaction between time and treatment arm will be included. Besides a random slope per participant, a random intercept and the inclusion of splines will be considered. In case complete observations are available for at least 80% of the participants, and if the pattern of missing observations is deemed noninformative, the mixed model will be compared to a generalized least squares model with unstructured, compound symmetry, or AR (1) covariance structures. The choice between the options will be made using the AIC criterion.

As multiple primary endpoints are measured repeatedly, multiple mixed models will be fit. For each of the models, the outcome of the global F or LR test (where appropriate) will be reported, and significance evaluated using the procedure of Benjamini-Hochberg.

The secondary outcome ‘feasibility in practice’, measured as the percentage of compliant participants, will not be tested, as there is no reference value for comparison.

### Cost-effectiveness analysis

The economic evaluation will consist of both a trial-based cost-effectiveness analysis with a time horizon of 1 year and a model-based cost-utility analysis with a lifetime horizon.

In the trial-based economic evaluation, the effects of the FMD will be compared to usual care and related to the difference in costs. Differences in mean costs and effects (HbA1c -level and QALYs) between strategies will be compared with two-sided bootstrapping. In a net-benefit analysis, costs will be related to the participant reported outcomes and presented in a cost-effectiveness acceptability curve. No discounting will be applied due to the time horizon of the economic evaluation of 1 year. Multiple imputation will be used for handling missing data. The evaluation will be performed from a healthcare and societal perspective. Sensitivity analysis will be carried out for the most important assumptions.

In the model-based cost-utility analysis the UKPDS Outcomes model will be used to estimate lifetime costs and QALYs for the FMD in comparison to usual care [32]. In this lifetime cost-utility analysis costs will be discounted at a percentage of 4% and effects at a percentage of 1.5%, according to the Dutch guidelines for health economic research [33]. Sensitivity analysis will be carried out for the most important input parameters.

Costs consist of healthcare costs and non-healthcare costs, such as costs of food (cost of the FMD and usual diet) and productivity costs (absence of paid and unpaid work). Healthcare costs concerns the costs of health care use related to diabetes during the year of follow-up e.g. GP visits, visits to dietician, medication. Costs of the FMD will be based on the putative market price. Health care use and absence from work will be assessed with the cost questionnaire (see Table 2). For the valuation of health care, standard prices published in the Dutch costing guidelines will be used [34]. Costs of absenteeism from paid work will be calculated using the friction cost method. Quality-of-life adjusted life-years (QALYs) will be estimated with aid of the EQ-5D-5L. Utilities will be calculated from the EQ-5D-5L questionnaire using the Dutch tariff [35]. Using the area-under-the-curve method for the utility scores obtained for each participant, the QALY outcome per participant will be obtained for the trial-based cost-effectiveness analysis. Furthermore, utility values will be used as input in the model for the lifetime cost-effectiveness analysis.

### Participant withdrawal

Participants who withdraw from the study will not be replaced. Participants will be asked to return at T4 and T8 for measurements of the primary and secondary endpoints, to be able to perform an intention-to-treat analysis. Subjects can leave the study at any time for any reason if they wish to do so without any consequences. The investigator can decide to withdraw a subject from the study for urgent medical reasons. All data generated up to the time of discontinuation from the study will be analysed and the reason for discontinuation (if known) will be documented.

### Safety

#### Adverse events

Previous experience with this FMD did not reveal serious side effects. Some weakness and headache were sometimes reported. Very few participants dropped out of previous studies because of physical side effects [20, 21]. Hypoglycaemia is not very likely to occur when participants use the FMD while also using metformin, since metformin has no effect on insulin secretion. Metformin will therefore be continued during the FMD. GPs are asked to inform the research team about Serious Adverse Events (SAEs) and Adverse Events (AEs). Participants will be asked at every visit about SAE’s. AE’s will be recorded at several study visits (see Table 2) and during telephone contacts with the research team.

#### Medication use

Medication dosage can be adapted by the GP at any moment according to the Dutch treatment guideline T2D for GP’s [23]. In the event of progression of the diabetes in the FMD group, other medication can be started by the GP in addition to metformin, for example a sulphonylurea derivative. When this occurs during the follow up, sulphonylurea derivatives will be stopped during the FMD, and an additional measurement of fasting glucose 1 week after the FMD will be necessary before the participant can restart this medication. GPs are asked to inform the research team when they start additional glucose lowering medication, so a personal safety plan can be developed for people in the FMD group.

#### Incidental findings

In the informed consent form, participants will give permission to be informed about incidental findings that have important consequences for their health. Their general practitioner will be informed likewise, and will then be asked to start up further diagnostic tests, treatment and/or referral to other specialists. Two examples of incidental findings are an unexpected laboratory result or unknown hypertension. MRI scans are not routinely evaluated by a radiologist for incidental findings. However, incidental findings can occur while performing the MRI scan or during analysis afterwards. Incidental findings that have important consequences for a participants’ health will be reported to the participant and the GP.

#### Monitoring

Monitoring will be done annually according to a monitor plan by internal monitors of the LUMC, since there is a negligible risk a data safety monitoring board will not be formed. A safety report will be submitted to the Medical Research Ethics Committee of the LUMC every year. An interim analysis will not be performed.

## Discussion

T2D can be greatly influenced by lifestyle interventions, including dietary interventions [3]. Improvement in metabolic parameters is certainly possible and may even lead to remission of T2D [6–8]. However, not everyone responds to a dietary intervention in the same way. Furthermore, severe restriction of calories for a longer period of time can be difficult to adhere to. A substantial proportion of people do not adhere to weight loss interventions [10]. Therefore, it is important to have several effective options for management of T2D, so people can find an option in accordance with their preferences. Previous studies have shown that FMDs can be beneficial in mice with hyperglycaemia [36] and healthy subjects [20, 21]. Studies in patients with T2D have not yet been performed.

The advantage of a FMD is that people have major dietary adjustment for a few days every month, and can thereafter return to a more ‘normal’ diet. In the FIT trial, participants do not get dietary advice for the time periods in between the FMD. However, dietary advice for patients with T2D is already part of usual care in general practices [23].

The FIT trial chose to only include T2D patients who are treated with lifestyle advice only or with lifestyle advice and a dose of metformin. T2D patients who use other types of antidiabetic medication, especially insulin, might be at risk for developing hypoglycaemia while using this medication in combination with the FMD. In the FIT trial, GPs and participants are instructed to contact the research team if additional medication is started, to be able to provide a personalised safety plan for the participant. Therefore, if in the FIT trial the use of the FMD improves metabolic parameters and/or reduces the need for medication, additional research is still necessary to assess the safety and effectivity in patients with T2D who use other types of antidiabetic medication.

To conclude, this randomised, controlled, assessor-blinded intervention trial will establish whether monthly cycles of a FMD improve metabolic parameters and/or reduce the need for medication in patients with T2D. In addition, many other health parameters will be measured. Furthermore, a cost-effectiveness evaluation will be performed and the feasibility in clinical practice will be measured as the percentage of compliant patients in the FMD group. Follow up is expected to be completed in June 2021.

## Data Availability

Not applicable for this study protocol. The datasets generated during the FIT trial are at the moment not publicly available due to the ongoing status of the study, but will be available upon completion of the data collection from the corresponding author on reasonable request.

## Abbreviations

AE: Adverse Event
ADF: Alternate Day Fasting
ALAT: Alanine aminotransferase
ASAT: Aspartate aminotransferase
BMI: Body mass index
CRP: C-reactive protein
eCRF: Electronic case report form
FIT: Fasting In diabetes Treatment
FMD: Fasting-mimicking diet
GCP: Good clinical practice
GP: General practitioner
HbA1c: Glycated haemoglobin
HDL: High-density lipoprotein
IGF-1: Insulin-like growth factor 1
IGF-BP-1: Insulin-like growth factor-binding protein 1
LDL: Low-density lipoprotein
LUMC: Leiden University Medical Center
MRI: Magnetic Resonance Imaging
OGTT: Oral glucose tolerance test
SAE: Serious Adverse Event
T2D: Type 2 diabetes
QALY: Quality-of-life adjusted life-years
