# Correction to: Fasting in diabetes treatment (FIT) trial: study protocol for a randomised, controlled, assessor-blinded intervention trial on the effects of intermittent use of a fasting-mimicking diet in patients with type 2 diabetes

**DOI:** 10.1186/s12902-020-00635-z

**Published:** 2020-11-04

**Authors:** Elske L. van den Burg, Marjolein P. Schoonakker, Petra G. van Peet, M. Elske. van den Akker-van Marle, Ko Willems van Dijk, Valter D. Longo, Hildo J. Lamb, Mattijs E. Numans, Hanno Pijl

**Affiliations:** 1 Department of Public Health and Primary Care, Leiden University Medical Center (LUMC), Postzone V0-P, Postbus 9600, 2300 RC Leiden, The Netherlands; 2 Department of Biomedical Data Sciences, Medical Decision Making, Leiden University Medical Center, Leiden, the Netherlands; 3 Internal Medicine, Leiden University Medical Center, Leiden, the Netherlands; 4 Human Genetics, Leiden University Medical Center, Leiden, the Netherlands; 5 FIRC Institute of Molecular Oncology, Milan, Italy; 6 Longevity Institute, Davis School of Gerontology, University of Southern California, Los Angeles, USA; 7 Radiology, Leiden University Medical Center, Leiden, the Netherlands

**Correction to: BMC Endocr Disord 20, 94 (2020)**

**https://doi.org/10.1186/s12902-020-00576-7**

Following publication of the original article [1], the authors identified the Table 1 is incorrect. The correct Table 1 is as below, and the original article has been corrected.

**Table 1:** Example meal plan of the fasting-mimicking diet for study participants

	Day 1	Day 2	Day 3	Day 4	Day 5
**Breakfast**	Tea	Tea	Tea	Tea	Tea
	Nut bar	Nut bar	Nut bar	Nut bar	Nut bar
	Algal Oil capsule				Algal Oil capsule
**Lunch**		Tea	Tea	Tea	Tea
	Tomato Soup	Mushroom Soup	Tomato Soup	Vegetable Soup	Tomato Soup
	Olives	Olives	Kale Crackers	Olives	Kale Crackers
	Kale crackers				
	Vitamin capsule	Vitamin capsule	Vitamin capsule	Vitamin capsule	Vitamin capsule
**Afternoon**	Tea	Tea	Tea	Tea	Tea
	Nut bar	Olives		Olives	
**Dinner**		Tea	Tea	Tea	Tea
	Minestrone Soup	Quinoa Mix Soup	Minestrone Soup	Quinoa Mix Soup	Minestrone Soup
	Choco crisp bar	Choco crisp bar		Choco crisp bar	
	Vitamin capsule	Vitamin capsule	Vitamin capsule	Vitamin capsule	Vitamin capsule
**During the day**		Syrup for water flavouring	Syrup for water flavouring	Syrup for water flavouring	Syrup for water flavouring
